# Catheterization Method and Functional Recovery of Neurogenic Bladder in Spinal Cord Injury

**DOI:** 10.1001/jamanetworkopen.2025.22030

**Published:** 2025-07-21

**Authors:** Carlos A. Aude, Devin M. Dishong, Arjun Menta, Jacob Jo, Jawad Khalifeh, Liam Hughes, Tej D. Azad, Arthur L. Burnett, Nicholas Theodore

**Affiliations:** 1 Department of Neurosurgery, Johns Hopkins University School of Medicine, Baltimore, Maryland; 2 Department of Urology, Johns Hopkins University School of Medicine, Baltimore, Maryland

## Abstract

**Importance:**

Neurogenic bladder dysfunction affects up to 80% of individuals with spinal cord injury (SCI), greatly impacting quality of life and health outcomes. Although current guidelines recommend intermittent catheterization primarily for infection prevention, its potential influence on recovery of volitional bladder control remains poorly understood.

**Objective:**

To determine whether intermittent catheterization, compared with indwelling catheterization, is associated with a higher likelihood of regaining volitional bladder control within 1 year after discharge in individuals with SCI, and to assess whether observed benefits are specific to bladder function or attributable to general neurological improvement.

**Design, Setting, and Participants:**

This retrospective cohort study used prospectively collected data from rehabilitation centers affiliated with the National Spinal Cord Injury Model Systems, representing diverse clinical settings across the US (2011-2021). Participants were adults (≥18 years) with traumatic SCI discharged with either indwelling or intermittent catheterization as primary bladder management. Data analysis was performed in October 2024.

**Exposure:**

Bladder management method at discharge (intermittent vs indwelling catheterization).

**Main Outcomes and Measures:**

The primary outcome was recovery of volitional bladder control at 1 year. Secondary outcomes included improvements in sacral motor and sensory function to assess the specificity of any observed benefits to bladder function. Propensity score matching was used to minimize selection bias. Multivariable logistic regression models were used to estimate adjusted odds ratios (aORs) for outcomes.

**Results:**

Among 1032 matched participants (median [IQR] age, 42 [27-58] years; 815 male [79.0%]), intermittent catheterization was associated with a higher rate of bladder control recovery than indwelling catheterization (88 patients [17.1%] vs 60 patients [11.6%]). After adjusting for matched covariates, intermittent catheterization was associated with significantly higher odds of bladder recovery (aOR, 2.11; 95% CI, 1.39-3.22; *P* < .001). No significant differences were observed for improvements in sacral motor function (120 patients [23.3%] vs 116 patients [22.4%]; aOR, 1.05; 95% CI, 0.59-1.90; *P* = .85) or sacral sensory function (127 patients [24.6%] vs 144 patients [27.9%]; aOR, 0.77; 95% CI, 0.43-1.37; *P* = .38), suggesting that the observed difference was specific to bladder function rather than general neurological recovery.

**Conclusions and Relevance:**

In this cohort study of patients with traumatic SCI, intermittent catheterization was independently associated with significantly higher odds of regaining volitional bladder control after SCI compared with indwelling catheterization, providing further evidence to support the prioritization of intermittent catheterization in this patient population.

## Key Points

**Question:**

Is the choice between indwelling and intermittent catheterization associated with the likelihood of regaining volitional bladder control after spinal cord injury, independently of general neurological recovery?

**Findings:**

In this propensity score–matched cohort study of 1032 adults with spinal cord injury, those managed with intermittent catheterization were more than twice as likely to regain bladder control within 1 year compared with those with indwelling catheters, with no differences in general neurological recovery. These findings were statistically significant.

**Meaning:**

The choice of catheterization method was significantly associated with bladder function recovery after spinal cord injury, supporting the prioritization of intermittent catheterization to optimize patient outcomes.

## Teaser

This cohort study examines whether indwelling vs intermittent catheterization is associated with the likelihood of regaining volitional bladder control within 1 year after discharge in adults with spinal cord injury.

## Introduction

Neurogenic bladder dysfunction is among the most prevalent and challenging sequelae of spinal cord injury (SCI), affecting up to 80% of individuals and profoundly diminishing their quality of life.^1,2^ This dysfunction can precipitate urinary tract infections, renal complications, and reduced functional independence.^3,4^ Consequently, the restoration of volitional bladder control stands as a critical rehabilitation goal, with important implications for patient autonomy, health outcomes, and overall well-being.^1,3^

The management of neurogenic bladder in patients with SCI primarily relies on 2 catheterization approaches: indwelling catheterization and intermittent catheterization.^4,5^ Indwelling catheterization allows continuous drainage, which may benefit those lacking sufficient hand function or requiring simplified care.^6^ However, long-term indwelling catheterization use elevates the risk of urinary tract infections and other adverse events, including bladder calculi and compromised detrusor muscle compliance.^7,8^ In contrast, intermittent catheterization periodically empties the bladder at prescribed intervals, simulating physiologic filling and voiding cycles and potentially preserving muscle function.^5,9^ Although current clinical guidelines recommend intermittent catheterization for reducing infection risk, its potential to restore volitional bladder control has not been sufficiently explored.^4,5,7^

Early investigations into the relationship between catheterization methods and functional bladder recovery were conducted in the 1970s and 1990s.^10,11,12,13,14^ However, their findings were constrained by small sample sizes and lack of control for confounding factors. Since these early efforts, the field has seen minimal systematic investigation of the relationship between catheterization method and bladder recovery, leaving a substantial gap in our understanding of optimal bladder management strategies for patients with SCI.

To address this research gap, we performed a multi-institutional, propensity score–matched analysis investigating whether the method of bladder management (intermittent vs indwelling catheterization) is associated with the likelihood of regaining volitional bladder control within 1 year after discharge in individuals with SCI. We also examined sacral motor and sensory improvements to discern whether any differences were specific to bladder recovery or reflected general neurological improvement. We hypothesized that intermittent catheterization use would be independently associated with higher odds of bladder recovery compared with indwelling catheterization. By controlling for multiple confounders in a large national database, we aimed to provide more definitive guidance on how catheterization practices may be associated with meaningful bladder recovery and better inform clinical decisions.

## Methods

### Data Source and Study Population

We conducted a retrospective cohort study using the National Spinal Cord Injury Model Systems (SCIMS) Database, a prospective, multi-institutional registry of individuals with SCI admitted to participating US centers since 1973.^15^ Our analysis included records from 2011 through 2021. Adults (aged ≥18 years) with traumatic SCI were eligible if they were discharged with indwelling or intermittent catheterization as their primary bladder management strategy and had documented follow-up 1 year after discharge. We excluded individuals who used alternative bladder management methods, had incomplete key variables or outcomes, or died within 1 year of discharge.

This study was conducted in accordance with the Strengthening the Reporting of Observational Studies in Epidemiology (STROBE) reporting guidelines. Because all data were deidentified and publicly available through the National Spinal Cord Injury Model Systems Database, this work qualified for exemption from Johns Hopkins Medicine institutional review board review and the need for informed consent under the Common Rule (45 CFR §46).

### Variables

The exposure variable for this study was the bladder management method at discharge, categorized as indwelling or intermittent catheterization. The primary outcome was regaining volitional bladder control at 1 year after discharge, as documented in the SCIMS database. In SCIMS, this variable indicates any self-reported or clinically observed partial or complete improvement that was sufficient to discontinue the previously used bladder management method. Secondary outcomes included sacral motor and sensory function improvements at 1 year after discharge. Sacral motor function was measured by the total American Spinal Injury Association (ASIA) motor index score for the S1 segment (range, 0-10 points, where a higher score denotes greater sacral motor function). Similarly, sacral sensory function was assessed by summing the ASIA sensory index scores for light touch and pinprick at S2 to S5 (range, 0-24 points, where a higher score denotes greater sacral sensory function). These sacral scores were chosen because the sacral segments are directly involved in the neural pathways controlling bladder function, providing a relevant measure of localized neurological recovery. Improvements in sacral motor and sensory scores were defined as any increase (≥1 ASIA point) from discharge to 1-year follow-up, treated as binary outcomes (improved vs not improved). This minimal threshold of 1 point was selected to maximize sensitivity in detecting neurological improvements.

Covariates included demographic characteristics (age at injury, sex, self-reported race [American Indian or Alaska Native, Asian or Pacific Islander, Black or African American, White, multiracial, or any other race not specified], self-reported ethnicity [Hispanic or not Hispanic], and body mass index [BMI], calculated as weight in kilograms divided by height in meters squared) and injury-related factors (year of injury, neurological level of injury, ASIA Impairment Scale grade at discharge, Functional Independence Measure score at discharge, and sacral motor and sensory scores at discharge). Data on race and ethnicity are included here because prior literature^16,17,18^ suggests that both initial bladder management choices and recovery after traumatic SCI differ by racial and ethnic group, making these variables potential confounders and key equity end points.

### Statistical Analysis

Descriptive statistics summarized baseline characteristics for unmatched and matched cohorts. Categorical variables were presented as frequencies and percentages, and continuous variables as medians and IQRs. The significance of differences between the cohorts across baseline characteristics was assessed using the Wilcoxon rank-sum test, Pearson χ^2^ test, or Fisher exact test where appropriate. To minimize confounding, we performed propensity score matching, generating scores via logistic regression that included all covariates. We then applied 1:1 nearest-neighbor matching with a caliper width of 0.15 SD of the propensity score, aiming for an absolute standardized mean difference (ASMD) of less than 0.1 across all matched variables. The selected caliper of 0.15 SD aimed to optimize covariate balance (ASMD <0.1) while avoiding excessive sample attrition, consistent with recommendations from the propensity score matching literature.^19^

Univariable analyses used the Fisher exact test to compare outcome proportions between the indwelling catheterization and intermittent catheterization groups. Multivariable logistic regression models estimated adjusted odds ratios (aORs) for outcomes, adjusting for matched covariates to account for residual confounding. Secondary analyses assessed sacral motor and sensory improvements to evaluate the specificity of bladder recovery. To accurately reflect the matched design and account for potential correlation within matched pairs, robust SEs were obtained by clustering participants by matched pairs.

All statistical analyses were conducted in October 2024 using R statistical software version 4.4.1 (R Project for Statistical Computing). Propensity score matching was implemented via the MatchIt package (version 4.6.0), whereas the sandwich package (version 3.1-1) was used to derive model estimates that are robust to heteroskedasticity and clustering. A 2-sided significance level of *P* < .05 was used for all tests.

## Results

### Descriptive Statistics

Before propensity score matching, 2827 individuals met the inclusion criteria: 2006 were managed with intermittent catheterization and 821 with indwelling catheterization. Table 1 provides a detailed breakdown of the prematching characteristics of the study cohort.

**Table 1.** Prematching Patient Characteristics, by Bladder Management Method

Characteristic	Patients, No. (%)			*P* value^a^
	Overall (N = 2827)	Intermittent catheterization (n = 2006)	Indwelling catheterization (n = 821)	
Demographics				
Age, median (IQR), y	36 (25-53)	33 (24-50)	45 (28-58)	<.001
Sex				
Female	542 (19.0)	332 (17.0)	210 (26.0)	<.001
Male	2284 (81.0)	1674 (83.0)	610 (74.0)	
Race				
American Indian or Alaska Native	22 (0.8)	12 (0.6)	10 (1.2)	.05
Asian or Pacific Islander	55 (1.9)	33 (1.6)	22 (2.7)	
Black or African American	608 (22.0)	449 (22.0)	159 (19.0)	
White	2023 (72.0)	1424 (71.0)	599 (73.0)	
Other race or multiracial^b^	119 (4.2)	88 (4.4)	31 (3.8)	
Ethnicity				
Hispanic	2456 (87.0)	1714 (85.0)	742 (90.0)	<.001
Not Hispanic	371 (13.0)	292 (15.0)	79 (9.6)	
Body mass index, median (IQR)^c^	25.7 (22.4-29.9)	25.5 (22.3-29.4)	26.3 (22.7-31.0)	<.001
Injury details				
Year of injury, median (IQR)	2015 (2013-2017)	2015 (2013-2017)	2015 (2013-2017)	.14
Level of injury, median (IQR)	4 (3-5)	4 (3-5)	4 (3-5)	
Cervical	1472 (52.0)	786 (39.0)	686 (84.0)	<.001
Thoracic	1141 (40.0)	1025 (51.0)	116 (14.0)	
Lumbar	210 (7.4)	192 (9.6)	18 (2.2)	
Sacral	4 (0.1)	3 (0.1)	1 (0.1)	
ASIA Impairment Scale grade at discharge				
A	1327 (47.0)	980 (49.0)	347 (42.0)	.002
B	475 (17.0)	313 (16.0)	162 (20.0)	
C	513 (18.0)	345 (17.0)	168 (20.0)	
D	512 (18.0)	368 (18.0)	144 (18.0)	
Functional Independence Measure total score, median (IQR)^d^	48 (27-65)	56 (37-68)	27 (19-39)	<.001
Sacral motor score, median (IQR)^e^	0 (0-2)	0 (0-2)	0 (0-3)	.15
Sacral sensory score, median (IQR)^f^	0 (0-10)	0 (0-10)	1 (0-10)	.06

Abbreviation: ASIA, American Spinal Injury Association.

^a^ *P* values were calculated with Wilcoxon rank sum test, Fisher exact test, or Pearson χ^2^ test.

^b^ Other race was documented when the self-identified racial group of the patient did not fit into any of the other classifications.

^c^ Body mass index is calculated as weight in kilograms divided by height in meters squared.

^d^ Score range is 0 to 91, with higher scores denoting greater physical independence in basic activities of daily living.

^e^ Refers to ASIA motor index score of S1 (gastrocnemius, soleus) at discharge (score range, 0-10, with higher scores denoting greater sacral motor function).

^f^ Refers to ASIA sensory index score of S2 to S5 (light touch plus pin prick) at discharge (score range, 0-24, with higher scores denoting greater sacral sensory function).

Significant differences were observed between the indwelling and intermittent catheterization groups across several key variables. Demographically, individuals using indwelling catheterization were significantly older, with a median (IQR) age of 45 (28-58) years compared with 33 (24-50) years in the intermittent catheterization group. In addition, a higher proportion of indwelling catheterization users vs intermittent catheterization users were female (210 patients [26.0%] vs 332 patients [17.0%]) and more likely to be Hispanic (292 patients [15.0%] vs 79 patients [9.6%]). Indwelling catheterization users were more likely to be White (599 patients [73.0%] vs 1424 patients [71.0%]), but this difference was not significant. There were also significant differences in BMI, with indwelling catheterization users having a higher median (IQR) BMI of 26.3 (22.7-31.0) compared with 25.5 (22.3-29.4) in the intermittent catheterization group. In terms of injury characteristics, indwelling catheterization users had a notably higher proportion of cervical injuries (686 patients [84.0%] vs 786 patients [39.0%]) and lower Functional Independence Measure total scores (median [IQR], 27 [19-39] vs 56 [37-68]) compared with intermittent catheterization users. The distribution of ASIA Impairment Scale grades differed significantly between the groups, with a higher proportion of intermittent catheterization users classified as ASIA Impairment Scale grade A (980 patients [49.0%] vs 347 patients [42.0%]).

Following matching, the final cohort consisted of 1032 individuals (median [IQR] age, 42 [27-58] years; 815 male [79.0%]), evenly split with 516 in each bladder management group. Table 2 presents the postmatching characteristics of the cohorts, revealing no significant differences between the indwelling and intermittent catheterization groups across all covariates (all *P* > .05 and ASMD <0.1).

**Table 2.** Postmatching Patient Characteristics, by Bladder Management Method

Characteristic	Patients, No. (%)			*P* value^a^	ASMD
	Overall (N = 1032)	Intermittent catheterization (n = 516)	Indwelling catheterization (n = 516)		
Demographics					
Age, median (IQR), y	42 (27-58)	42 (27-58)	43 (27-58)	.50	0.030
Sex					
Female	217 (21.0)	103 (20.0)	114 (22.0)	.40	0.021
Male	815 (79.0)	413 (80.0)	402 (78.0)		
Race					
American Indian or Alaska Native	8 (0.8)	4 (0.8)	4 (0.8)	>.99	0.000
Asian or Pacific Islander	26 (2.5)	12 (2.3)	14 (2.7)		0.004
Black or African American	196 (19.0)	96 (19.0)	100 (19.0)		0.008
White	769 (75.0)	385 (75.0)	384 (74.0)		0.002
Other race or multiracial^b^	33 (3.2)	19 (3.7)	14 (2.7)		0.010
Ethnicity					
Hispanic	937 (91.0)	466 (90.0)	471 (91.0)	.60	0.010
Not Hispanic	95 (9.2)	50 (9.7)	45 (8.7)		
Body mass index, median (IQR)^c^	26.1 (22.6-30.1)	26.3 (22.9-29.9)	25.9 (22.2-30.5)	.60	0.006
Injury details					
Year of injury, median (IQR)	2015 (2013-2017)	2014 (2013-2017)	2015 (2013-2017)	.70	0.024
Level of injury, median (IQR)	4 (3-5)	4 (3-5)	4 (3-5)		
Cervical	844 (82.0)	419 (81.0)	425 (82.0)	.70	0.012
Thoracic	153 (15.0)	78 (15.0)	75 (15.0)		0.006
Lumbar	34 (3.3)	19 (3.7)	15 (2.9)		0.008
Sacral	1 (<0.1)	0	1 (0.2)		0.002
ASIA Impairment Scale grade at discharge					
A	433 (42.0)	220 (43.0)	213 (41.0)	>.99	0.014
B	192 (19.0)	96 (19.0)	96 (19.0)		0.000
C	207 (20.0)	102 (20.0)	105 (20.0)		0.006
D	200 (19.0)	98 (19.0)	102 (20.0)		0.008
Functional Independence Measure total score at discharge, median (IQR)^d^	29 (20-44)	30 (20-45)	28 (20-42)	.30	0.082
Sacral motor score, median (IQR)^e^	0 (0-4)	0 (0-3)	0 (0-4)	.40	0.045
Sacral sensory score, median (IQR)^f^	2 (0-11)	1 (0-11)	2 (0-12)	.60	0.037

Abbreviations: ASIA, American Spinal Injury Association; ASMD, absolute standardized mean difference.

^a^ *P* values were calculated with Wilcoxon rank sum test, Fisher exact test, or Pearson χ^2^ test.

^b^ Other race was documented when the self-identified racial group of the patient did not fit into any of the other classifications.

^c^ Body mass index is calculated as weight in kilograms divided by height in meters squared.

^d^ Score range is 0 to 91, with higher scores denoting greater physical independence in basic activities of daily living.

^e^ Refers to ASIA motor index score of S1 (gastrocnemius, soleus) at discharge (score range, 0-10, with higher scores denoting greater sacral motor function).

^f^ Refers to ASIA sensory index score of S2 to S5 (light touch plus pin prick) at discharge (score range, 0-24, with higher scores denoting greater sacral sensory function).

### Outcome Analyses

In our primary analysis of the matched cohort, 88 patients (17.1%) in the intermittent catheterization group regained volitional bladder control by 1 year after discharge, compared with 60 patients (11.6%) in the indwelling catheterization group (*P* = .02, Fisher exact test). In the multivariable logistic regression, intermittent catheterization users had more than twice the odds of regaining bladder control compared with indwelling catheterization users (aOR, 2.11; 95% CI, 1.39-3.22; *P* < .001).

Although the primary outcome demonstrated an improvement for the intermittent catheterization group in regaining bladder control, secondary analyses assessing general neurological improvement showed no significant differences between the groups. Sacral sensory improvement occurred in 127 patients (24.6%) in the intermittent catheterization group and 144 patients (27.9%) in the indwelling catheterization group (aOR, 0.77; 95% CI, 0.43-1.37; *P* = .38), whereas sacral motor improvement was noted in 120 patients (23.3%) in the intermittent catheterization group and 116 patients (22.4%) in the indwelling catheterization group (aOR, 1.05; 95% CI, 0.59-1.90; *P* = .85). These results are summarized in Table 3.

**Table 3.** Primary and Secondary Outcomes by Bladder Management Group

Outcome	Patients, No. (%)		Adjusted OR (95% CI)^a^	*P* value
	Intermittent catheterization (n = 516)	Indwelling catheter (n = 516)		
Primary outcome^b^				
Regained bladder control	88 (17.1)	60 (11.6)	2.11 (1.39-3.22)	<.001
Secondary control outcomes^c^				
Sacral motor improvement	120 (23.3)	116 (22.4)	1.05 (0.59-1.90)	.85
Sacral sensory improvement	127 (24.6)	144 (27.9)	0.77 (0.43-1.37)	.38

Abbreviation: OR, odds ratio.

^a^ OR was estimated using multivariable logistic regression models adjusted for demographic and clinical factors and clustered SEs to account for matching.

^b^ Discontinuation of bladder management method occurred because of sufficient improvement in bladder function.

^c^ Refers to 1-point or greater improvement in American Spinal Injury Association score at the S1 (motor) or S2-S5 (sensory) neurological levels.

## Discussion

In this multi-institutional, propensity score–matched cohort analysis, we observed that intermittent catheterization was associated with more than double the odds of regaining volitional bladder control compared with indwelling catheterization. The specificity of this association with bladder function, evidenced by the absence of significant differences in sacral motor or sensory improvements between groups, suggests that the method of bladder management may play a distinct role in functional recovery. These findings substantially advance our understanding beyond early observational studies^10,11,12,13,14^ that were limited by small sample sizes and inadequate control of confounding variables.

Several physiological mechanisms may contribute to the differences in bladder outcomes observed in this study. Indwelling catheterization prevents the physiologic filling and stretching needed to maintain bladder compliance, thus inducing detrusor atrophy, which gradually compromises volitional control.^20,21,22,23^ Conversely, intermittent catheterization preserves a more natural bladder-filling pattern, potentially preventing muscle atrophy, maintaining afferent signaling, and supporting neuroplastic adaptations critical for recovering volitional function.^8,20,21^

Chronic inflammation further contributes to the differences observed between intermittent and indwelling catheterization. Prolonged exposure to a catheter, as occurs with indwelling catheterization use, disrupts the protective mucosal barrier of the urinary tract and increases the risk of infection and inflammation.^7,24^ This chronic inflammation can lead to tissue remodeling and fibrosis, further diminishing bladder function and reducing elasticity.^25^ Intermittent catheterization, by minimizing catheterization time, reduces the risk of infection and inflammation, thus promoting a healthier bladder environment that is more conducive to functional recovery.^8,26^

Furthermore, the regular bladder activity associated with intermittent catheterization might support neuroplasticity by maintaining afferent signaling from the bladder to the spinal cord, which is crucial for re-establishing coordinated detrusor-sphincter function.^9,25,26^ Indwelling catheterization, by bypassing these natural bladder-filling and emptying sensations, disrupts afferent signaling and may interfere with the ability of the spinal cord to adapt and reorganize, impeding volitional bladder control recovery. The benefits of intermittent catheterization thus seem to extend beyond its role in preventing infection by potentially influencing the neural pathways governing bladder function and promoting neuroplasticity that supports recovery of volitional control. These findings also complement related research efforts demonstrating the positive effects of neurostimulation and activity-based interventions on bladder function recovery.^22,27,28,29,30^

These findings have immediate implications for clinical practice. The apparent association between intermittent catheterization and functional bladder recovery, combined with its known advantages in preventing urinary tract infections, strongly support its selection as the preferred method of bladder management when feasible. However, implementation requires careful consideration of individual patient factors, including hand function, cognitive status, and caregiver support. Medical teams should engage in shared decision-making with patients about the potential long-term benefits of intermittent catheterization for functional recovery, moving beyond the traditional focus on infection prevention. Our results also suggest the need to re-evaluate current clinical guidelines. Although existing recommendations favor intermittent catheterization primarily for infection prevention,^4,5^ our findings potentially indicate that functional bladder recovery should be considered a distinct and important benefit. Guidelines should be updated to reflect the potential role of catheterization method on recovery of volitional control, particularly during the critical first year after injury when neuroplasticity may be heightened.

Future research should focus on prospective, randomized clinical trials comparing intermittent and indwelling catheterization with standardized urodynamic assessments to assess causal relationships. Mechanistic studies exploring biological pathways affected by different catheterization methods, such as inflammation markers and neuroplasticity indicators, are necessary to elucidate the underlying processes driving bladder function recovery. Investigating adjunctive therapies that enhance neuroplasticity alongside intermittent catheterization could further improve bladder function recovery outcomes.^27,28,29,30^

### Limitations

Despite leveraging a large, multi-institutional dataset and rigorous propensity-matched design, our study has several notable limitations. First, owing to its retrospective, observational nature, we cannot definitively establish causality, and residual confounding likely persists. Although we adjusted extensively for demographic and injury-related characteristics, the potential for residual confounding remains because the database lacked granular details on potentially influential comorbidities—such as metabolic or neurological conditions—that could influence both catheterization selection and bladder recovery outcomes. In addition, variability in clinical practice across centers, including differences in caregiver support, institutional protocols, or patient preferences, might have introduced biases despite our efforts to balance measured covariates.

Second, our primary outcome was based on self-reported or clinically observed discontinuation of the initial catheterization strategy owing to recovery of volitional bladder control. This outcome lacks the objectivity provided by urodynamic assessment and may fail to capture partial improvements or the necessity of additional voiding interventions. For example, participants could continue to rely on compression techniques, voiding schedules, or other supportive methods that are not recorded in the registry. Moreover, SCIMS did not record pharmacological therapies (eg, anticholinergic medications) or urological procedures (eg, bladder augmentation). Thus, we cannot exclude the possibility that medical or surgical interventions contributed to catheter discontinuation and affected bladder outcomes independently of catheterization methods.

Third, although the SCIMS database encompasses multiple specialized SCI centers, it may not comprehensively represent broader clinical practice—particularly nonspecialized settings, facilities treating patients with less-severe or nontraumatic injuries, or regions with limited access to advanced interventions. This limitation affects the generalizability of our results and may obscure clinical practice management complexities, including transitions between catheterization methods or combination approaches used by patients over time.^31^ Prospective studies using standardized urodynamic assessments, extended follow-up, and detailed collection of pharmacological, surgical, and intermediate bladder recovery data are warranted to clarify these nuances and further refine optimal bladder management strategies following traumatic SCI.

## Conclusions

Intermittent catheterization was associated with a more than 2-fold increase in the odds of recovering volitional bladder control within 1 year after injury, independently of broader neurological improvements. These findings provide evidence in favor of prioritizing intermittent catheterization in clinical practice, emphasizing its potential to aid in functional bladder recovery beyond its known role in infection prevention. Clinicians should consider adopting intermittent catheterization as the preferred bladder management method when feasible to optimize outcomes and improve the quality of life for individuals with spinal cord injury.

## Notes

Supplement.
Data Sharing Statement
