# Supplementary material for: Catheterization Method and Functional Recovery of Neurogenic Bladder in Spinal Cord Injury
Source: JAMA Netw Open. 2025 Jul 21;8(7):e2522030. doi: 10.1001/jamanetworkopen.2025.22030 (PMC12281237; doi:10.1001/jamanetworkopen.2025.22030)
Supplement: Supplement. — Data Sharing Statement [file jamanetwopen-e2522030-s001.pdf]

Data Sharing Statement

Aude. Catheterization Method and Functional Recovery of Neurogenic Bladder in Spinal Cord Injury. *JAMA Netw Open*. Published July 21, 2025. doi:10.1001/jamanetworkopen.2025.22030

Data
Data available: No

Additional Information
Explanation for why data not available: This study was conducted using data provided by the SCIMS under a grant from the National Institute on Disability, Independent Living, and Rehabilitation Research (NIDILRR grant number 90SIMS0016). NIDILRR is a Center within the Administration for Community Living (ACL), Department of Health and Human Services (HHS). The contents of this publication do not necessarily represent the policy of NSCISC, NIDILRR, ACL, or HHS and should not be considered an endorsement by the Federal Government. De-identified data collected before September 1, 2021, are publicly available for download and stripped of all HIPAA-defined identifiers. For further information on accessing SCIMS data, please contact the National Spinal Cord Injury Statistical Center (NSCISC).
